# Extra vitamin D from fortification and the risk of preeclampsia: The D-tect Study

**DOI:** 10.1371/journal.pone.0191288

**Published:** 2018-01-25

**Authors:** Maria Stougaard, Peter Damm, Peder Frederiksen, Ramune Jacobsen, Berit Lilienthal Heitmann

**Affiliations:** 1 Research Unit for Dietary Studies at the Parker Institute and Department of Clinical Epidemiology, Bispebjerg og Frederiksberg Hospital, The Capital Region, Copenhagen, Denmark; 2 Department of Obstetrics, Rigshospitalet and Institute of Clinical Medicine, Faculty of Health and Medical Sciences, University of Copenhagen, Copenhagen, Denmark; 3 Research Unit for Chronic Conditions, Bispebjerg og Frederiksberg Hospital, The Capital Region, Copenhagen, Denmark; 4 National Institute of Public Health, University of Southern Denmark, Copenhagen, Denmark; 5 Section for General Practice, Department of Public Health, University of Copenhagen, Copenhagen, Denmark; Univesity of Iowa, UNITED STATES

## Abstract

The objective of the study was to examine if exposure to extra vitamin D from food fortification was associated with a decrease in the risk of preeclampsia. The study was based on a natural experiment exploring the effect of the abolition of the Danish mandatory vitamin D fortification of margarine in 1985. The effect of the extra vitamin D (1.25μg vitamin D/100 g margarine) was examined by comparing preeclampsia risk in women who have been exposed or unexposed to extra vitamin D from the fortified margarine during pregnancy, and who gave birth in the period from June 1983 to August 1988. The Danish National Patient Registry allowed the identification of pregnancies complicated by preeclampsia. The study included 73,237 women who gave birth during 1983–1988. We found no association between exposure to vitamin D fortification during pregnancy and the risk of any of the pregnancy related hypertensive disorders, including preeclampsia: Odds ratios (OR, 95%) for all hypertensive pregnancy related disorders among exposed vs. unexposed women was (OR 1.04, 95%CI: 0.98,1.10). In conclusion, the extra vitamin D from the mandatory vitamin D fortification did not influence the risk of preeclampsia.

## Introduction

Preeclampsia is a severe pregnancy related syndrome characterized by hypertension and proteinuria with onset after the 20^th^ week of pregnancy. It affects approximately 3–5% of all pregnant women. Preeclampsia may present as or develop into hemolysis, elevated liver enzyme levels, and low platelet levels (HELLP) syndrome or eclampsia, characterized by seizures and other severe complications [1, 2]. Preeclampsia and its complications elevate risk of morbidity and mortality for both mother and the newborn child [3]. Several factors have been shown to be associated with an elevated preeclampsia risk: young and old maternal age, null parity, multiple pregnancies, obesity, family history of preeclampsia, autoimmune disorders and preexisting hypertension or diabetes [1, 4, 5]. Immune system-mediated dysfunctions in placental development, leading to poor perfusion and maternal endothelia damage, further resulting in hypertension and proteinuria, have been suggested to be involved in the pathogenesis of preeclampsia [6]. Nevertheless, the exact mechanisms leading to the onset of the syndrome are yet poorly understood [1].

Vitamin D deficiency is quite common throughout life including during pregnancy [7–9], where the need for vitamin D is elevated, especially during the third trimester [10]. Prevalence of vitamin D deficiency and insufficiency among Danish pregnant women in their third trimester has in a recent study been shown to reach 6.3% and 19.2%, respectively [11]. The classical vitamin D functions are related to bone health and calcium homeostasis. Today, however, it is well-established that vitamin D also have many important non-skeletal roles, including those related to the immune system [12, 13]. Vitamin D deficiency has been linked to immune dysregulation and endothelial dysfunction mechanisms also involved in the pathogenesis of preeclampsia [14]. The systematic reviews and meta-analyses of results including observational studies on vitamin D and preeclampsia so far have reported conflicting results [15–18]. Reviews and meta-analyses of randomized controlled trials (RCT) examining the effects of the vitamin D supplementation during pregnancy concluded that vitamin D supplementation did not lead to a decreased risk of preeclampsia [19, 20].

The main objective of the present study was to investigate if pregnant women who received small, but consistent extra vitamin D doses from food fortification had a lower risk of developing preeclampsia than pregnant women who did not receive this extra vitamin D.

## Methods and materials

### National data sources

Our study was conducted utilizing the unique Danish national administrative and medical registers, collecting information on individual level from the entire population. The Danish Civil Registration System (CSR), established in 1968, records all people living in Denmark with a 10-digit civil person register (CPR) number, enabling linkage of individual information from different nationwide registers and large clinical databases [21]. Thus, we retrieved our study population and its demographic information from the CSR. The Danish Medical Birth Registry (MBR), established in 1973, records information from the antenatal care visits and deliveries of all women with permanent residence in Denmark [22]. From the MBR we retrieved the usual pregnancy-related information of the women as well as information on their newborns. The Danish National Patient Registry (DNPR), established in 1977, records information on all patients discharged from Danish non-psychiatric hospitals; from 1995 emergency and outpatient departments visit-related information is also included into the registry [22]. From the DNPR we retrieved information on gestational hypertension and preeclampsia diagnoses.

### Study design and population

**Fig 1:**
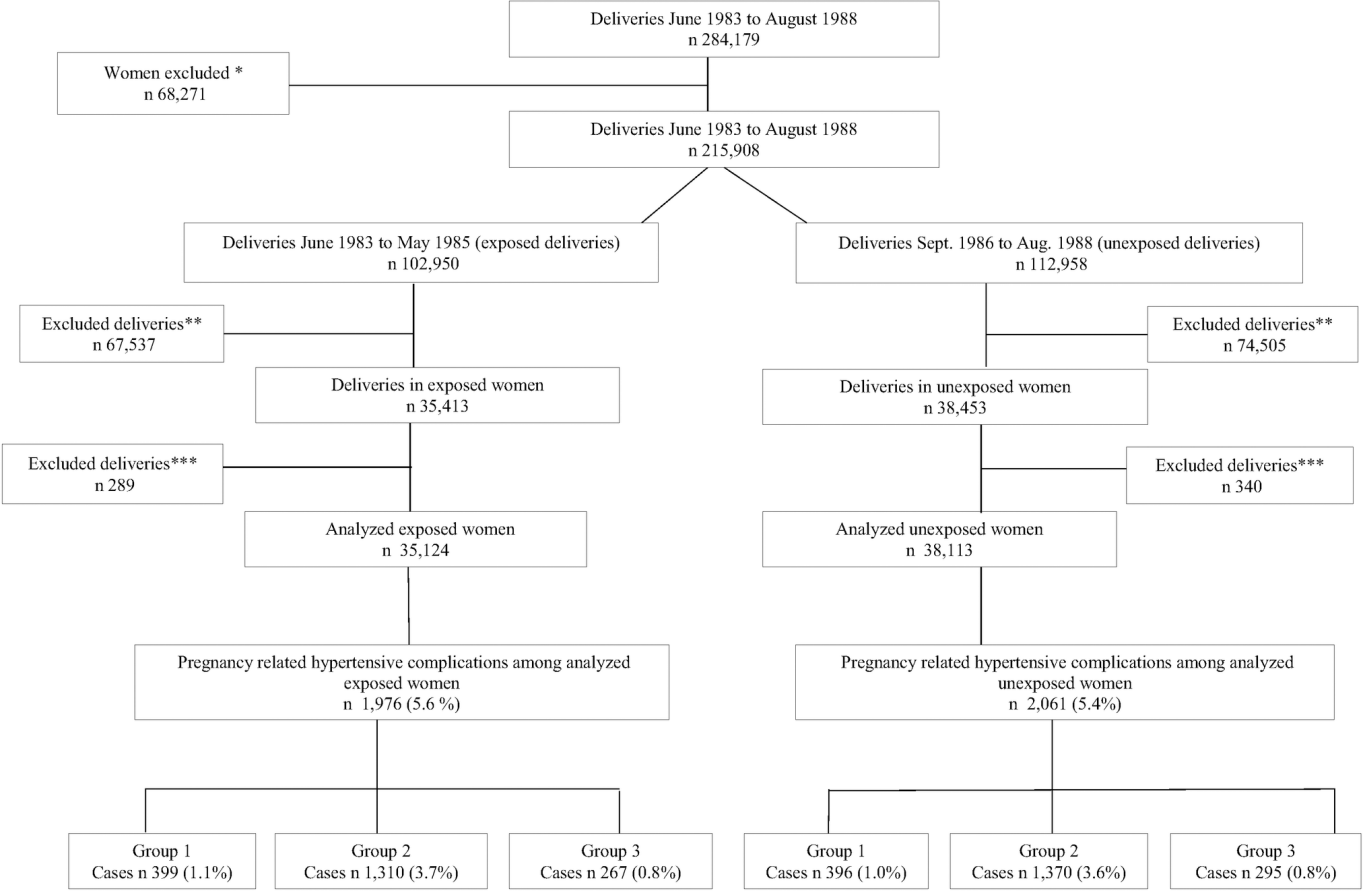
Flow chart of the study population. *Deliveries excluded during the 15 months’ wash-out period from June 1985 to August 1986. **Births which were not the women first, misclassification of the offspring’s birth weight, or gestational weeks was < 22 weeks. ***Excluded due to missing information on maternal age at delivery, singleton and multiple births, gestational age at delivery, birth weight or offspring gender. ^1^Group: Gestational hypertension (code 63700). ^2^Group: Mild preeclampsia (code 63703), Preeclampsia, unspecified (code 63709) & Toxemia (63799). ^3^Group: Severe preeclampsia (code 63704), Eclampsia (code 63719).

A Danish fortification policy that required adding of 1.25μg of vitamin D per 100g margarine, corresponding to approximately 13% (3–29%) of the daily vitamin D intake [23], was terminated June 1^st^ 1985 [24–26]. This change in national legislation concerning vitamin D fortification provided us with a natural experiment and a unique opportunity to differentiate Danish women, who during their pregnancies, were or were not exposed to the extra vitamin D from the fortified margarine. Thus, into our study we initially included all women in Denmark who gave birth in the period from the 1^st^ of June 1983 to the 31^st^ of August 1988—in total 284,179 births. Then a 15 months’ ‘wash-out period’ (consisting of a full 9 months of pregnancy and additional 6 months, securing that the fortified margarine was no longer available at home or in stores) was introduced and lasted from the 1^st^ of June 1985 to the 31^st^ of August 1986; the 68,271 births that occurred during the wash-out period were excluded. Consequently, 102,950 births from the 1^st^ of June 1983 to the 31^st^ of May 1985, comprised the exposed group; and 112,958 births from the 1^st^ of September 1986 to the 31^st^ of August 1988 comprised the unexposed group. The study population was further restricted only to include nulliparous women who gave birth after 22 completed gestational weeks. In addition, we excluded births with misclassification of the offspring’s birth weight, as well as those with missing information on maternal age at delivery, gestational age, single or multiple pregnancy, birth weight or gender of offspring. Thus, the final study population consisted of 35,124 pregnancies among exposed nulliparous women, and 38,113 pregnancies among unexposed nulliparous women. A flow chart of the study population is illustrated in Fig 1.

### Definition of outcome

Until 1994, the disease diagnoses in DNPR were coded according to the International Statistical Classification of Diseases and Related Health Problems 8^th^ Revision (ICD-8). In our study, the following ICD-8 codes defined the outcome: gestational hypertension (code 63700), mild preeclampsia (code 63703), unspecified preeclampsia (code 63709), severe preeclampsia (code 63704), toxemia (code 63799), and eclampsia (code 63719). The included diagnoses where categorized into three groups (Table 1). A woman could only be assigned one diagnosis; if a woman had more than one of the defined diagnoses, she was grouped according to the diagnosis code indicating the most severe diagnosis of the included outcomes.

**Table 1:** Definition of gestational hypertension, preeclampsia and eclampsia by ICD8 codes.

Grouping	ICD8 codes with description
Group 1	Gestational hypertension (code 63700)
Group 2	Mild preeclampsia (code 63703) Preeclampsia, unspecified (code 63709) Toxemia (63799)
Group 3	Severe preeclampsia (code 63704) Eclampsia (code 63719)

### Variables

As pregnancies with multiple fetuses are associated with a higher risk of preeclampsia [3], the included pregnancies were categorized as either singletons or multiple pregnancies.

As offspring male gender has been shown to be associated with increased maternal risk of preeclampsia [27], gender of the offspring, male vs. female, was considered in the analyses.

As the ultraviolet (UV) B radiation from the sun during autumn and winter months in Denmark is too weak to induce synthesis of vitamin D in the skin [28, 29], month of the delivery was also considered in our analyses. Months of delivery were categorized into 4 seasons: November to January (Winter); February to April (Spring); May to July (Summer) and August to October (Fall). This categorization was based on the 25(OH)D distribution seen in 7,437 whites form the British Birth Cohort [28] where 25(OH)D levels were highest in September, started declining in October, were lowest in January through April, and then started increasing from May [28].

### Statistical analyses

Differences in demographic characteristics of the exposed and unexposed women were tested using Chi-squared test for categorical variables and Mann-Whitney rank-sum test for continuous variables.

Logistic regression was used to investigate association between exposure status and the risk of preeclampsia. The model for all the outcomes, as well as the models differentiated by outcome severity (Table 1) was run. First, crude odds ratio (OR) and corresponding 95% confidence intervals (CI) were calculated (crude model). Subsequently, we adjusted for potential confounders (i.e. maternal age at delivery, single or multiple pregnancy, season of birth and offspring gender). Further, we hypothesized that the effect of fortification will be heterogeneous with respect to season of birth, so that preeclampsia risk will be highest for unexposed women delivering during winter and spring months, where vitamin D synthesis from the sun is restricted. To test that, the interaction between exposure status and season of delivery was tested using likelihood ratio test.

All analyses were performed using Stata version 13 StataCorp LP,College Station, Texas, USA; (www.stata.com); p < 0.05 was considered statistically significant.

### Ethics

The Danish Data Protection Agency (J.no. 2012-41-1156) approved data access and retrieval from the registries. As the study was based on already collected data, approval from the ethical committee was not required.

## Results

Overall, a total of 1,976 (5.6%) and 2,061 (5.4%) of the women, in the exposed and unexposed groups, respectively, developed a pregnancy related hypertensive syndrome; the majority of the developed diagnoses were mild or unspecified preeclampsia as well as toxemia (Fig 1). The descriptive characteristic of the women by exposure status are shown in Table 2. The distribution of hypertensive syndromes by their severity between the exposed and unexposed women did not differ (p = 0.2). Unexposed women were older than the exposed women (p<0.0001).Other characteristics of the unexposed and exposed women or their offspring did not differ.

**Table 2:** Characteristics of women and their offspring, according to whether or not the women were exposed (N = 35,124) or unexposed (N = 38,113) to extra vitamin D from fortification during pregnancy.

Characteristics	Exposed, n(%)	Unexposed, n(%)	p-value†
**Maternal age at delivery**			<0.0001
< 20	3,065(8.7)	2,546(6.7)	
20–24.9	15,458(44.0)	15,793(41.4)	
25–30.9	12,551(35.7)	14,724(38.6)	
30–35.9	3,284(9.4)	4,136(10.9)	
≥ 35	766(2.2)	914(2.40)	
**Outcome**			
Gestational hypertension^1^	399(1.1)	396(1.0)	0.2
Mild–unspecified preeclampsia Toxemia^2^	1,310(3.7)	1,370(3.6)	0.3
Severe preeclampsia and eclampsia^3^	267(0.8)	295(0.8)	0.8
All Cases	1,976(5.6)	2,061(5.4)	0.2
**Season of delivery**			0.2
November-January	8,096(23.0)	8,859(23.3)	
February—April	8,979(25.6)	9,381(24.6)	
May—July	9,152(26.1)	10,071(26.4)	
August—October	8,897(25.3)	9,802(25.7)	
**Singleton or multiple pregnancy**			0.9
Singleton	34,784(99.0)	37,748(99.0)	
Multiple	340(1.0)	365(1.0)	
**Gender of new-born**			0.07
Female	17,063(48.6)	18,263(47.9)	
Male	18,061(51.4)	19,850(52.1)	

^1^ICD-8 code: 63700

^2^ICD-8 code: 63703, 63709 and 63799

^3^ICD-8 code: 63704 and 63719

^†^ tested by X^2^-test, or Mann-Whitney rank-sum test

Both the crude and adjusted models showed no association between exposure status and mild, unspecified preeclampsia or toxemia. Likewise there were no associations between the exposure status and gestational hypertension or severe preeclampsia and eclampsia (Table 3). The detailed analysis of the adjusted models showed that women who gave birth during summer and fall (May to October) had the lowest risk of mild–unspecified preeclampsia, or toxemia (Table 4). The models testing interaction between exposure status and season of preeclampsia diagnosis did not reveal any consisting findings.

**Table 3:** Crude and adjusted odds ratio for the risk of pregnancy related hypertensive disorders among women exposed vs unexposed to extra vitamin D during their pregnancy.

Outcomes	n cases	Crude model OR (95%CI)	Adjusted model OR (95%CI)†
Gestational hypertension^1^	795	1.09(0.95,1.26)	1.11(0.97,1.28)
Mild preeclampsia Preeclampsia, unspecified Toxemia^2^	2,680	1.04(0.96,1.12)	1.03(0.95,1.11)
Severe preeclampsia and eclampsia^3^	562	0.98(0.83,1.16)	0.98(0.83,1.16)
All above	4,037	1.04(0.98,1.11)	1.04(0.98,1.10)

^†^Adjusted for maternal age at delivery, singleton or multiple pregnancy, gender of offspring and season of delivery

^1^ICD-8 code: 63700

^2^ICD-8 code: 63703, 63709 and 63799

^3^ICD-8 code: 63704 and 63719

**Table 4:** Covariates in the adjusted models for the risk of pregnancy related hypertensive disorders among women exposed vs. unexposed to extra vitamin D during their pregnancy.

	Gestational hypertension	Mild and unspecified preeclampsia or toxemia	Severe preeclampsia and eclampsia	All cases
	Adj. OR (95% CI)^†^	Adj. OR (95% CI)^†^	Adj. OR (95% CI)^†^	Adj. OR (95% CI)^†^
Unexposed	Reference	Reference	Reference	Reference
Exposed	1.11(0.97,1.28)	1.03(0.95,1.11)	0.98(0.83,1.16)	1.04(0.98,1.10)
Maternal age at delivery < 20	Reference	Reference	Reference	Reference
20–24.9	1.83(1.28,2.61)	0.96(0.83,1.11)	1.04(0.75,1.45)	1.06(0.94,1.21)
25–30.9	1.85(1.29,2.66)	0.81(0.70,0.94)	1.00(0.72,1.40)	0.95(0.83,1.08)
30–35.9	2.35(1.59,3.49)	0.76(0.63,0.92)	1.06(0.71,1.59)	0.98(0.84,1.14)
≥ 35	3.52(2.17,5.71)	0.83(0.62,1.11)	1.58(0.92,2.72)	1.23(0.99,1.54)
Singleton	Reference	Reference	Reference	Reference
Multiple	1.13(0.58,2,18)	2.07(1.55,2.77)	4.70(3.10,7.13)	2.32(1.84,2.93)
Offspring gender Male	Reference	Reference	Reference	Reference
Offspring gender Female	0.93(0.80,1.07)	0.88(0.81,0.95)	0.96(0.82,1.14)	0.90(0.84,0.95)
Season of delivery Nov.–Jan.	Reference	Reference	Reference	Reference
Season of delivery Feb.–Apr.	1.09(0.89,1.34)	0.95(0.85,1.05)	0.89(0.70,1.14)	0.96(0.88,1.06)
Season of delivery. May–July	1.04(0.84,1.27)	0.87(0.78,0.98)	1.06(0.84,1.33)	0.93(0.85,1.01)
Season of delivery Aug.–Oct.	1.06(0.88,1.30)	0.86(0.77,0.96)	1.08(0.85,1.36)	0.92(0.84,1.01)

^†^ Adjusted for maternal age at delivery, singleton or multiple pregnancy, gender of offspring, and season of delivery

## Discussion

We examined if the Danish mandatory fortification program adding 1.25 μg of vitamin D to 100 g margarine influenced the risk to develop preeclampsia in more than 70,000 Danish women with first time pregnancies. We were not able to detect any association between the exposure to extra vitamin D from the fortified margarine and the risk to develop a pregnancy-related hypertensive complication, including preeclampsia. However, we observed a seasonality pattern in the incidence of hypertensive disorders which suggests that vitamin D from the sun may protect against preeclampsia.

Literature on vitamin D and preeclampsia is extensive [15–18, 20, 30–32]. The association was examined in observational studies, where exposure to vitamin D was assessed by both vitamin D intake and serum 25(OH)D concentrations, and in vitamin D supplementation trials. A systematic review of 11 observational studies identified slightly more studies with no association [30]. Another systematic review and meta-analysis [15] including 9 observational studies reported a significant association between low maternal serum 25(OH)D level and preeclampsia risk (OR 1.79, 95%CI:1.25,2.58) [15]. A recent Cochrane review examining the effect of vitamin D supplementation on risk of preeclampsia included two RCTs with in total 219 pregnant women, receiving at least 10 μg of vitamin D per day in the intervention groups or placebo [33, 34]. The meta-analysis of the results from these trials showed not significant trend towards a lower risk of preeclampsia among women who had received the vitamin D supplementation: average risk ratio (RR) 0.52, 95% CI: 0.25, 1.05 [31].

The amount of vitamin D added to the fortified margarine in Denmark was 1.25μg vitamin D/100g. Based on previous studies we estimated that the included pregnant women could have received 0.4–0.6 micrograms of extra vitamin D per day from the fortified margarine [20, 33–35]. Thus, the amount of extra vitamin D from fortified margarine in our study was about 20 times lower than in the trails, and the extra vitamin D added to the fortified margarine might have been too low to exert protective effects on preeclampsia. Moreover, recent RCT supplementing pregnant women with 10 versus 110 μg vitamin D/day found no effect of the supplementation [36]. This trial also demonstrated that the women with optimal vitamin D levels at trial entry, coinciding with pregnancy planning, had a lower risk of preeclampsia [36]. Specifically, the trial suggested that for supplementation to be effective, the levels of 25(OH)D (i.e. vitamin D biomarker in the blood) at conception had to be at least 40ng/ml [36]. To achieve this level, a vitamin D intake of at least 100 μg/day, or significant sunshine exposure, is required [37]. Another trial by Sasan and colleagues [38] similarly reported that supplementing with vitamin D early in pregnancy was essential in the prevention of preeclampsia. The reasons why vitamin D, either from fortification, supplementation or sun, may be more important at conception than later in pregnancy are possibly related with the vitamin D immunomodulatory effects during placental implantation.

Our results indicated possible seasonality in diagnosis of mild preeclampsia cases: women delivering in May to October had a slightly lower risk of mild preeclampsia compared to those delivering November to January. This finding calls for more detail seasonality investigations in our data. The seasonality in the preeclampsia diagnoses has been examined in several previous studies. A Swedish study including 10,666 pregnant women found a lower risk of preeclampsia during summer (June to September) compared to deliveries during winter, spring and fall [39]. Similar results were found in a Norwegian study including 1,869,388 pregnant women [40]. Though various seasonal factors (e.g. infections) may be operating [41, 42], the seasonality pattern in preeclampsia incidence may specifically indicate the role of vitamin D deficiency in preeclampsia development: dermal synthesis of vitamin D is possible only when the skin is exposed to solar UVB radiation, which is not strong enough to induce vitamin D synthesis in countries at high latitudes during winter. The prevalence of pre-eclampsia was found to behighest (3.08%) in December. The prevalence declined during spring and summer to the lowest level (2.46%) in August, and increased gradually in the autumn months (Table 1). The prevalence ratio for pre-eclampsia was 1.25 for December, with August as the reference month (Table 1).

The observation that women who were unexposed to extra vitamin D from fortification were slightly older than the exposed women reflect the development, that over time Danish women are getting older when having their first child [43].

### Methodological considerations

The distinct feature of our study was utilization of the natural experiment. We examined preeclampsia incidence in women who were exposed or unexposed to vitamin D fortification, and were pregnant and consequently gave birth, during a narrow period of time: from 1983 to 1988. The design was built on the assumption that no other major changes, except for the abolition of the mandatory vitamin D fortification of margarine, occurred during 1983–1988. We were not able to identify other major societal or environmental events that occurred during 1983–1988. However, self-reported data on the Danish population diet indicated a change towards a more healthy diet during 1985–2001 [44, 45]. The food disappearance statistic, on the other hand, showed minimal changes in margarine consumption: from 16.9 kg/inhabitant in 1983, to 16.2 kg/inhabitant in 1985 and 17.0 kg/inhabitant in 1988 [46]. Notably, food disappearance statistic is based on purchase and not actually consumption, and dietary surveys are dependent on self-reporting, where underreporting bias in relation to fat intake has been shown to increase in the period 1985–2001 [45].

The strength of our study lies in the use of the Danish register data. The Danish national administrative and health registers are characterized by good quality and validity and are being used extensively for research purposes [22, 47, 48].

## Conclusion

The present study found no evidence to support that the extra vitamin D from a mandatory food fortification program resulted in a lower preeclampsia risk. Nevertheless, the observed seasonality pattern in preeclampsia incidence indicates that vitamin D from the sun may have a role in preeclampsia prevention, and such a possibility needs to be examined in further studies.
